# A Meta-Comparison of the Effects of High-Intensity Interval Training to Those of Small-Sided Games and Other Training Protocols on Parameters Related to the Physiology and Performance of Youth Soccer Players

**DOI:** 10.1186/s40798-019-0180-5

**Published:** 2019-02-21

**Authors:** Philipp Kunz, Florian Azad Engel, Hans-Christer Holmberg, Billy Sperlich

**Affiliations:** 1 Integrative & Experimental Exercise Science and Training, Institute of Sport Science, University of Würzburg, Judenbühlweg 11, 97082 Würzburg, Germany; 2 Institute of Sport and Sport Science, Heidelberg University, Heidelberg, Germany; 3 School of Sport Sciences, UiT The Arctic University of Norway, Tromsø, Norway; 4 Swedish Winter Sports Research Centre, Department of Health Sciences, Mid Sweden University, Östersund, Sweden

**Keywords:** Adolescents, Children, Conditioning, Endurance, Repeated sprint

## Abstract

**Background:**

High-intensity interval training (HIIT) is frequently employed to improve the endurance of various types of athletes. To determine whether youth soccer players may benefit from the intermittent load and time efficiency of HIIT, we performed a meta-analysis of the relevant scientific literature.

**Objectives:**

Our primary objective was to compare changes in various physiological parameters related to the performance of youth soccer players in response to running-based HIIT to the effects of other common training protocols (i.e., small-sided games, technical training and soccer-specific training, or high-volume endurance training). A secondary objective was to compare specifically running-based HIIT to a soccer-specific form of HIIT known as small-sided games (SSG) in this same respect, since this latter type of training is being discussed extensively by coaches.

**Method:**

A systematic search of the PubMed, SPORTDiscus, and Web of Science databases was performed in August of 2017 and updated during the review process in December of 2018. The criteria for inclusion of articles for analysis were as follows: (1) comparison of HIIT to SSG or some other training protocol employing a pre-post design, (2) involvement of healthy young athletes (≤ 18 years old), and (3) assessment of variables related to endurance or soccer performance. Hedges’ g effect size (*d*_ppc2_) and associated 95% confidence intervals for the comparison of the responses to HIIT and other interventions were calculated.

**Results:**

Nine studies, involving 232 young soccer players (mean age 16.2 ± 1.6 years), were examined. Endurance training in the form of HIIT or SSG produced similar positive effects on most parameters assessed, including peak oxygen uptake and maximal running performance during incremental running (expressed as *V*_max_ or maximal aerobic speed (MAS)), shuttle runs (expressed as the distance covered or time to exhaustion), and time-trials, as well as submaximal variables such as running economy and running velocity at the lactate threshold. HIIT induced a moderate improvement in soccer-related tests involving technical exercises with the soccer ball and other game-specific parameters (i.e., total distance covered, number of sprints, and number of involvements with the ball). Neuromuscular parameters were largely unaffected by HIIT or SSG.

**Conclusion:**

The present meta-analysis indicates that HIIT and SSG have equally beneficial impacts on variables related to the endurance and soccer-specific performance of youth soccer players, but little influence on neuromuscular performance.

## Key Points

- Various reports on responses of both youth and adults to high-intensity interval training (HIIT) have been published, but no systematic comparison of the effects of HIIT and other training regimes on parameters related to the performance of youth soccer players has yet been published.
- The calculated effect sizes indicate that HIIT has more beneficial effects than various other exercise programs on all of the parameters examined, with the exceptions of sprinting and jumping performance and repeated sprint ability.
- Small-sided games (SSG), a sport-specific form of HIIT, exerted similar effects on parameters related to soccer performance (i.e., maximal oxygen uptake, maximal running performance, running economy, and running at the lactate threshold).
- HIIT and SSG both enhance the key performance of youth soccer players in a time-efficient manner, leaving more time for training sport-specific skills such as technique and tactics.

## Background

Youth soccer is characterized by constant changes in the intensity of a variety of activities, including standing, walking, running, and sprinting with frequent changes in direction, as well as jumping, often with involvement of the ball and/or opponents [1]. This varying intensity (from low to high), as well as matches lasting as long as 90 min (depending on age), involves on average > 80% of peak heart rate [2] and approximately 75% of maximal oxygen uptake [1] in youth soccer players, imposing considerable demands on both aerobic and anaerobic energy production (i.e., in connection with short sprints, jumps, tackles) [1, 3]. Accordingly, high-level endurance performance is an important prerequisite for success [3].

High-volume or continuous low-intensity training has been employed successfully to improve certain aspects of endurance performance, such as peak oxygen uptake (VO_2peak_), individual anaerobic threshold, and/or maximal velocity in youth soccer players [4, 5]. HIIT, characterized by periods of intense exercise at > 90% of peak heart rate (HR_peak_) alternating with periods of low-intensity activity [6], and SSG, a soccer-specific form of HIIT [7], show great potential to improve certain aspects of the endurance of youth soccer players. Both of these types of training improve key variables related to success in soccer, e.g., VO_2peak_ [8, 9] and maximal running performance (i.e., shuttle run testing and time-trials) [10, 11], as well as performance in connection with soccer-specific tests (i.e., total distance covered, number of sprints, and number of contacts with the ball) [12, 13] in a time-efficient manner [14].

The lengths of training periods and intervals of rest, number of intervals and sessions of HIIT or SSG per week, and the number of players and size of the pitch can be varied in virtually an endless number of ways [14–18]. Various HIIT protocols improve different indicators of endurance performance [10] and SSG enhances both fitness and soccer-specific performance [14, 16]. Clearly, in addition to technical and tactical skills, such physiological factors are also important determinants of success [19].

From a practical point of view, questions arise among coaches concerning (1) the overall responses of youth soccer players to HIIT in comparison to other endurance protocols with respect to endurance and other important determinants of performance, such as the ability to sprint and jump; and (2) whether SSG, a sport-specific form of HIIT, results in improvement of these same parameters comparable to those achieved with running-based HIIT.

Our aim here was to systematically compare the effects of HIIT on variables related to the performance by youth soccer players as reported in numerous publications during the past decade to those of other training regimes (in particular SSG). Such analysis should aid in designing HIIT and/or SSG to improve the endurance of youth soccer players most effectively.

## Methods

### Databases and Search Profile

This systematic analysis of peer-reviewed investigations on the effects of HIIT and SSG on several parameters related to performance in youth soccer players was conducted in accordance with established guidelines [20]. A comprehensive computerized search of the PubMed, SPORTDiscus, and Web of Science databases, with no restriction as to year of publication, was performed in August of 2017, with an update during the review process in December of 2018, employing the following search strings: high-intensity interval training OR high-intensity training OR intensive interval training OR aerobic interval training OR sprint interval training OR specific endurance training OR aerobic endurance OR aerobic training AND young players OR young athletes OR adolescent athletes OR teen athletes OR junior athletes OR children athletes OR children OR adolescents AND soccer OR football.

**Fig. 1:**
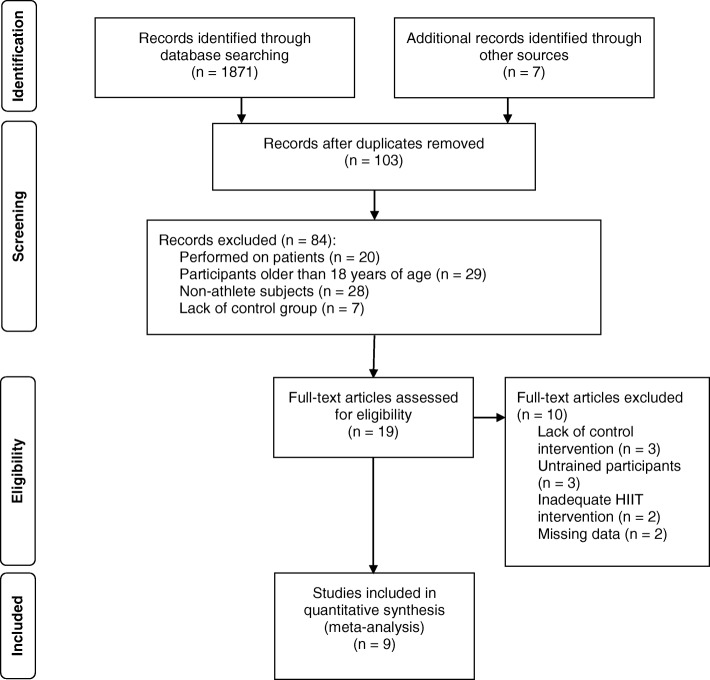
Selection of the articles to be analyzed, from initial identification to inclusion

The search was limited to original research articles written in English and published in peer-reviewed journals. The screening and selection process is illustrated in Fig. 1.

In addition, the references cited by the articles retrieved were examined for potential relevance. Data was extracted from the studies included by one of the authors and this extraction checked independently by a second author.

### Selection and Quality Assessment of Articles

The inclusion criteria were as follows: (1) involvement of endurance training in the form of HIIT (e.g., at ≥ 90% of maximal oxygen uptake [21], 90–95% of HR_peak_ [10], or as (supra)maximal interval sprinting [22]); (2) involvement of male soccer players 18 years of age or younger; (3) pre- and post-assessment of exercise performance, physiological parameters related to performance, and/or soccer-specific performance; (4) intervention for at least 4 weeks; and (5) inclusion of a control treatment (i.e., SSG, high-volume training, or technical training).

Studies on patients with specific conditions (e.g., obesity, diabetes mellitus, or asthma) were excluded, as were conference abstracts, dissertations, theses, and articles that had not undergone peer review.

To ensure the methodological quality of the articles selected, the criteria of the Physiotherapy Evidence Database (PEDro) scale were applied, with one point for each criterion fulfilled and a maximal possible score of 10 [23]. The quality of each article is documented in Table 1.

**Table 1:** The Physiotherapy Evidence Database (PEDro) score for each article included

	Item											
Article	1^a^	2	3	4	5	6	7	8	9	10	11	Total
Los Arcos et al. (2015) [27]	1	1	1	1	0	0	0	1	1	1	1	7
Faude et al. (2013) [4]	1	1	1	1	0	0	0	0	1	1	1	6
Faude et al. (2014) [29]	1	1	1	1	0	0	0	0	1	1	1	6
Helgerud et al. (2001) [29]	1	1	1	1	0	0	0	1	1	1	1	7
Hill-Haas et al. (2009) [11]	1	1	1	1	0	0	0	0	1	1	1	6
Impellizzeri et al. (2008) [12]	1	1	1	1	0	0	0	1	1	1	1	7
Impellizzeri et al. (2006) [28]	1	1	1	1	0	0	0	0	1	1	1	6
Siegler et al. (2013) [30]	1	0	0	1	0	0	0	1	1	1	1	5
Sperlich et al. (2011) [10]	1	1	1	1	0	0	0	1	1	1	1	7

^a^This item was not included when calculating the PEDro score

### Statistical Analyses

The findings on HIIT were compared to those with (1) all of the other interventions, (2) all of the other interventions except SSG, and (3) SSG alone.

Effect sizes (ES) were calculated as the mean difference between the experimental and control groups, divided by SD_pooled_, as recommended by Carlson and Schmidt [24].

$$ \mathrm{SDpooled}=\sqrt{\frac{\left({n}_{\mathrm{exp}}-1\right){\mathrm{SD}}_{\mathrm{exp}}^2+\left({n}_{\mathrm{contr}}-1\right){\mathrm{SD}}_{\mathrm{contr}}^2}{n_{\mathrm{exp}}+{n}_{\mathrm{contr}}-2}} $$

where *n* is the number of participants in the experimental (*n*_exp_) and control groups (*n*_cont_) and SD_exp_ and SD_cont_ the corresponding pretest standard deviations.

In addition, in light of the tendency for findings with small samples to be positively biased, leading to overestimation, a correction factor (CP) was employed:

$$ \mathrm{CP}=1-\frac{3}{4\times \left({n}_{\mathrm{exp}}+{n}_{\mathrm{contr}}-2\right)-1} $$

This approach to the calculation of ES (*d*_ppc2_) is optimal in the case of studies involving pre- and post-testing and a control group [25]:

$$ {d}_{\mathrm{ppc}2}=\mathrm{CP}\ \Big[\frac{\left({M}_{\mathrm{post},\exp }-{M}_{\mathrm{pre},\exp}\right)-\left({M}_{\mathrm{post},\mathrm{contr}}-{M}_{\mathrm{pre},\mathrm{contr}}\right)}{{\mathrm{SD}}_{\mathrm{pooled}}} $$

The ES values obtained were classified as trivial (< 0.10), small (0.10–0.30), moderate (0.30–0.50), or large (> 0.50) [26]. Heterogeneity was assessed using an *I*^2^ value and the 95% confidence interval (CI) calculated.

All statistical analyses were carried out in version 11.5.1.0 of the Med-Calc software (MedCalc Software, Mariakerke, Belgium).

## Results

### Characteristics of the Studies Analyzed

Nine studies were included (see Table 2 for a summary) and assessed in accordance with the PEDro scale, resulting in an average score of 6.3 (range 5–7). In four of these, SSG was the control program [11, 27–29], the five others comparing either a form of high-volume training [4, 10, 30] or technical soccer training [12, 13] to HIIT.

Altogether, these studies involved 232 male soccer players (mean 22.6 ± 8.2 participants per study; range 15–39), 13–18 years of age (mean age: 16.2 ± 1.6 years), of whom 50% performed intense interval training and the other 50% control programs of exercise, i.e., either SSG, some form of low-intensity high-volume exercise, or soccer-specific drills (e.g., generic soccer training, technical and tactical training).

**Table 2:** The design of the studies included in this meta-analysis and characteristics of their participants

Reference (year)	Subjects /age (years)	Total number of sessions/duration/duration of one intervention session/duration of one control session	Intervals of exercise (number and duration)	Interval intensity	Duration of rest	Intensity of exercise during rest	VO_2peak/max_ before the intervention (mL·kg^−1^ · min^−1^)	VO_2peak/max_ after the intervention (mL·kg^−1^ · min^−1^)	Change in VO_2max_ (%)	Findings concerning the HIIT
Los Arcos et al. (2015)^1^ [27]	15/16	12/6 weeks/ 25 min/25 min	HIIT							MAS ↔; CMJ ↔
			3 × 4 min	90–95% HR_max_	3 min	50–60% HR_max_	n.i.	n.i.	n.i.	
			SSG							
			3 × 4 min	n.i.	3 min	n.i.	n.i.	n.i.	n.i.	
Faude et al. (2014)^2^ [29]	19/17	8/4 weeks/ 22 min/36 min	HIIT							IAT ↑; CMJ ↓ DJ ↔; *V*_peak_ ↓ Lac_max_ ↔ 5-m, 10-m, 30-m sprint ↔; CinD ↔
			2 × (12–15 × 15 s)	40% above IAT	10 min/15 s	n.i.	n.i.	n.i.	n.i.	
			SSG							
			4 × 4 min	n.i.	4 min (PR)	n.i.	n.i.	n.i.	n.i.	
Faude et al. (2013)^2^ [4]	20/16	12–15/5.5 weeks/ 33 min/47 min	HIIT							IAT ↑; CMJ ↓ DJ ↓; *V*_max_ ↔
			2 × (12–15 × 15–30 s)	25–40% above IAT	10 min (AR)/15–30 s	n.i.	n.i.	n.i.	n.i.	
			HVT							
			30–60 min	80–95% IAT	–	–	n.i.	n.i.	n.i.	
Sperlich et al. (2011)^1^ [10]	19/13	13/5 weeks/ 28.8 min/57.3 min	HIIT							VO_2max_ ↑; 1000-m run ↑; 20-m, 30-m, 40-m sprint ↑; DJ, SJ, CMJ ↔
			4–15 × 30 s–4 min	90–95% HR_max_	1–3 min	50–60% HR_max_	55.1 ± 4.9	58.9 ± 4.7	+ 6.9	
			HVT							
			6–30 min	50–70% HR_max_	–	–	55.3 ± 4.3	56.4 ± 3.7	+ 2.0	
Hill-Haas et al. (2009)^1^ [11]	19/15	14/7 weeks/ 66 min/66 min	HIIT (intervals, RSA, COD, sprints)							VO_2max_ ↔; YYIRTL1 ↑; MSFT ↔; TTE ↔; 5-m, 20-m sprint ↔; RSA ↔
			–	–	–	n.i.	60.2 ± 4.6	61.4 ± 3.5	+ 2.0	
			SSG							
			2–3 × 6–13 min	n.i.	1–3 min	n.i.	59.3 ± 4.5	58.9 ± 5.5	− 0.7	
Impellizzeri et al. (2008)^1^ [12]	21/18	11/4 weeks/ 25 min/25 min	HIIT							VO_2max_ ↑; LSPT total performance ↔; LSPT total performance fatigued ↑
			4 × 4 min	90–95% HR_max_	3 min (AR)	n.i.	~ 56.6	~ 58.9	~ + 4	
			TT							
			–	–	–	–	~ 57.7	~ 57	~ − 1.2	
Impellizzeri et al. (2006)^1^ [28]	29/17	16/8 weeks/ n.i./n.i.	HIIT							VO_2max_ ↑; IAT ↑; RE at LT ↔; VO_2_ at LT ↑; % VO_2max_ at LT ↑; Ekblom’s test ↑
			4 × 4 min	90–95% HR_max_	3 min	60–70% HR_max_	59.7 ± 4.1	60.2 ± 3.9	+ 0.8	
			SSG							
			4 × 4 min	90–95% HR_max_	3 min	60–70% HR_max_	61.4 ± 4.6	61.8 ± 4.5	+ 0.7	
Siegler et al. (2003)^1^ [30]	34/16	30/10 weeks/ n.i./n.i.	Functional HIIT (sprinting and jumping)							LIST ↑; 20-m sprint ↑; VJ ↔
			3–5 × 4–6 reps.	100%	n.i.	n.i.	n.i.	n.i.	n.i.	
			VOT							
			n.i.	n.i.	n.i.	n.i.	n.i.	n.i.	n.i.	
Helgerud et al. (2001)^1^ [13]	19/18	18/8 weeks/ 28 min/30 min	HIIT							VO_2max_ ↑; IAT ↑ ↔; VO_2_ at LT ↑; 10-m, 40-m sprint ↔; VJ ↔
			4 × 4 min	90–95% HR_max_	3 min	50–60% HR_max_	58.1 ± 4.5	64.3 ± 3.9	+ 10.7	
			TT							
			–	–	–	–	58.4 ± 4.3	59.5 ± 4.4	+ 1.9	

*↑* significant positive effect; ↓ significant negative effect; ↔ no significant effect

^1^Controlled pre-post-design

^2^Cross-over study design

*AR* active rest, *CinD* change-in-direction run, *CMJ* countermovement jump, *DJ* drop jump, *HR*_*max*_ maximal heart rate, *HVT* high-volume training, *IAT* individual anaerobic threshold, *Lac*_*max*_ maximal blood lactate concentration, *LIST* Loughborough Intermittent Shuttle Test, *LSPT* Loughborough Soccer Passing Test, *LT* lactate threshold, *MAS* maximal aerobic speed, *MSFT* multistage fitness test, *n.i.* not indicated, *PR* passive rest, *Reps* repetitions, *RE* running economy, *RSA* repeated sprint ability, *SJ* squat jump, *SSG* small-sided game, *TT* technical training, *TTE* time to exhaustion, *V*_*max/peak*_ maximal velocity during incremental step test, *VJ* vertical jump, *VO*_*2max*_ maximal oxygen uptake, *VOT* volume-oriented training at low intensities, *VT* ventilatory threshold, *YYIETL1* Yo-Yo intermittent endurance test level 1, *YYIRTL1* Yo-Yo intermittent recovery test level 1

All of these participants played regularly for a club, in most cases professional. Four studies [11, 12, 27, 28] explicitly excluded goalkeepers, with their special physical requirements and training regimes, from their interventions. In five of these nine studies in which this parameter was measured [10–13, 28], the initial mean VO_2peak_ of the subjects was 57.4 ± 1.7 (range 55.1–60.2) mL·kg^−1^·min^−1^. HR_max_ was determined with incremental treadmill tests [10–13, 28], incremental field tests [4, 29], or the University of Montreal Test (UM-TT) [27].

### Protocols of the HIIT Interventions

The mean duration of the interventions was 6.4 ± 2.0 weeks (range 4–10), with an average of 2.9 ± 0.9 sessions per week (range 2–4.5).

The HIIT protocols employed differed with respect to intensity, duration, and work-to-rest ratio (Table 2). Notably, three studies involved 4 × 4 min of running at 90–95% of maximal heart rate (HR_max_), with 3-min intervals of rest [12, 13, 28].

In most cases, the target intensity was > 90% HR_max_ [10, 12, 27, 28, 30], although two studies set this intensity at 25–40% greater than the participant’s individual anaerobic threshold velocity [4, 29]. Two studies employed elements of functional HIIT, such as repeated squats, jumps, interval sprints, and agility drills [11, 30]. The intervals of exercise were either short (~ 15–30 s; [4, 29] or 4 min in length [12, 13, 27, 28], with one study involving a mixture (30 s–4 min) [10]. The recovery period between intervals of exercise ranged from 15 to 180 s.

### The SSG Interventions

In four cases, HIIT was compared to SSG [11, 27–29]. Two of these studies employed 4 × 4-min games [28, 29], one 3 × 4-min of SSG [27], and one 2–3 sets of 6–13-min games [11]. The recovery between intervals of exercise ranged from 1 to 4 min and the pitch dimensions varied, as did the duration and number of players (range 2 against 1 to 7 against 7).

### Maximal or Peak Oxygen Uptake

#### Comparison of HIIT to All Other Interventions

**Fig. 2:**
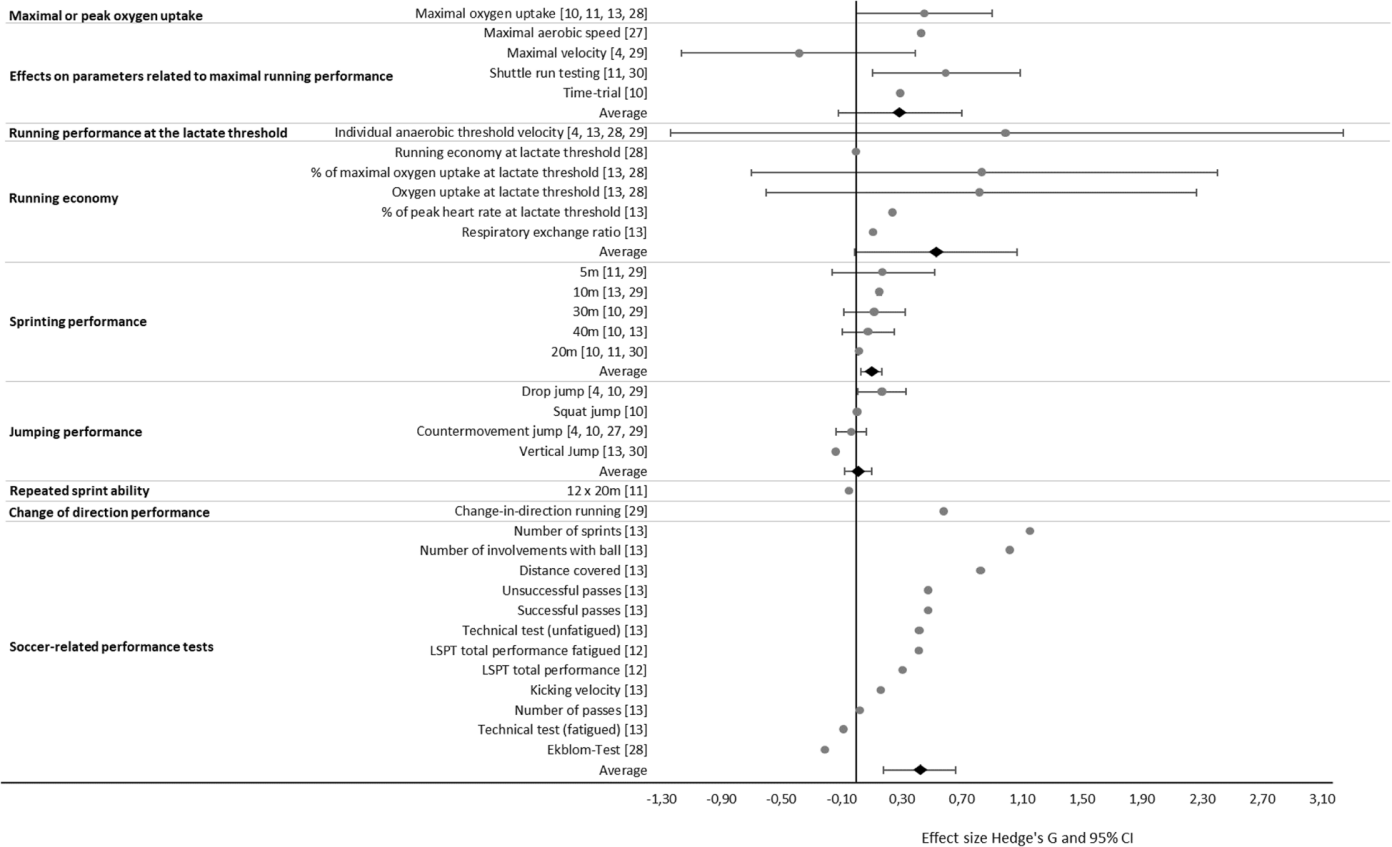
Comparison of the overall and individual effect sizes (*d*_ppc2_, dots) and corresponding 95% confidence intervals (lines) for parameters related to the performance of youth soccer players with HIIT to those of all other interventions

In comparison to all other interventions, HIIT induced a moderate mean positive effect on peak oxygen uptake (mean *d*_ppc2_ 0.45 ± 0.46; range 0.08–1.11). Among the four analyses of peak or maximal oxygen uptake, one effect was trivial positive [28], one small positive [11], one moderate positive [10], and one large positive [13] (Fig. 2).

#### Comparison of HIIT to All Other Interventions Except SSG

**Fig. 3:**
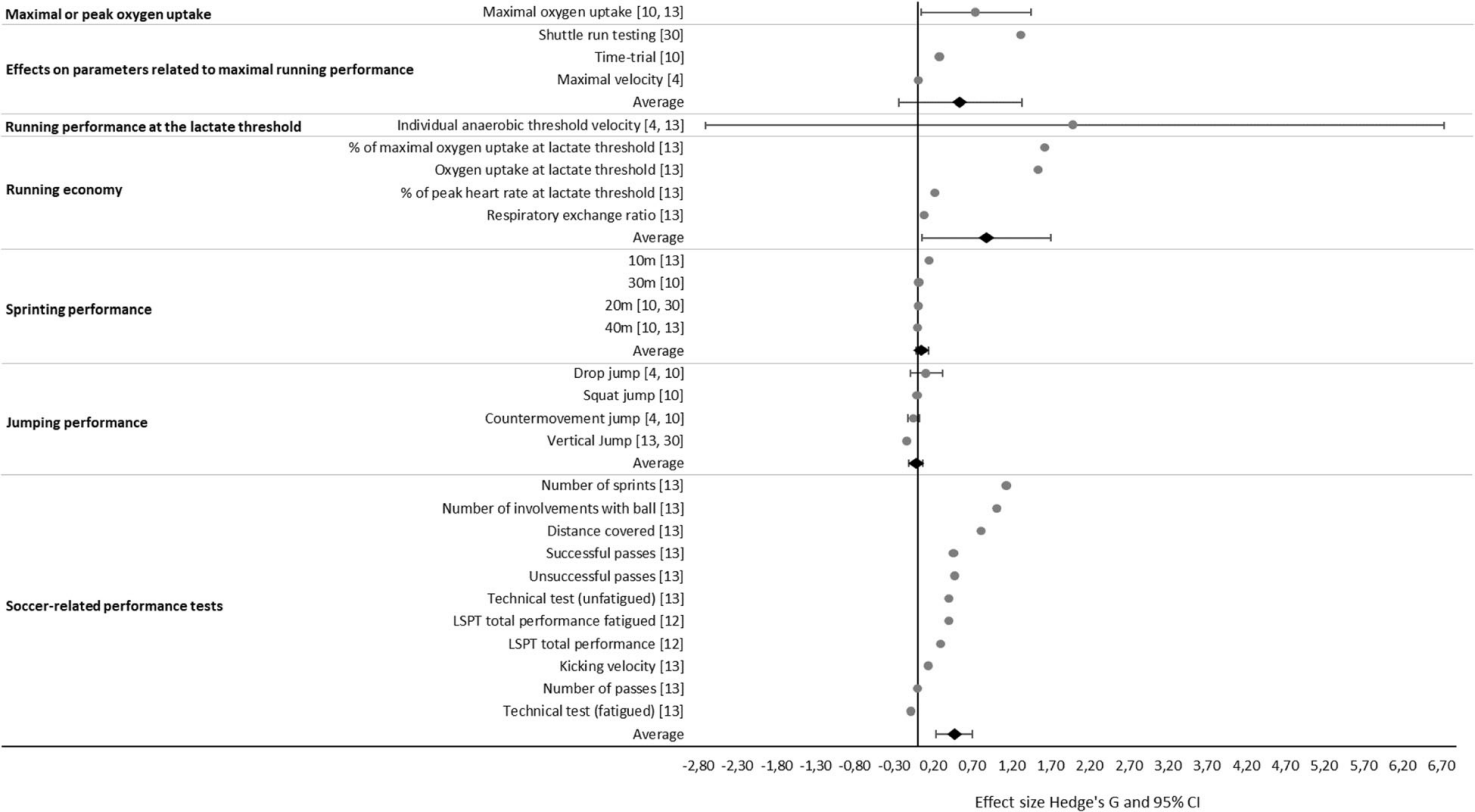
Comparison of the overall and individual effect sizes (*d*_ppc2_, dots) and corresponding 95% confidence intervals (lines) for parameters related to the performance of youth soccer players with HIIT to those of all other interventions except SSG

In comparison to all interventions except SSG, HIIT resulted in a large mean positive effect on maximal or peak oxygen uptake (mean *d*_ppc2_ 0.75 ± 0.50; range 0.40–1.11). In the two studies analyzed in this respect, one reported a moderate positive [10] and the other a large positive effect [13] (Fig. 3).

#### Comparison of HIIT to SSG

**Fig. 4:**
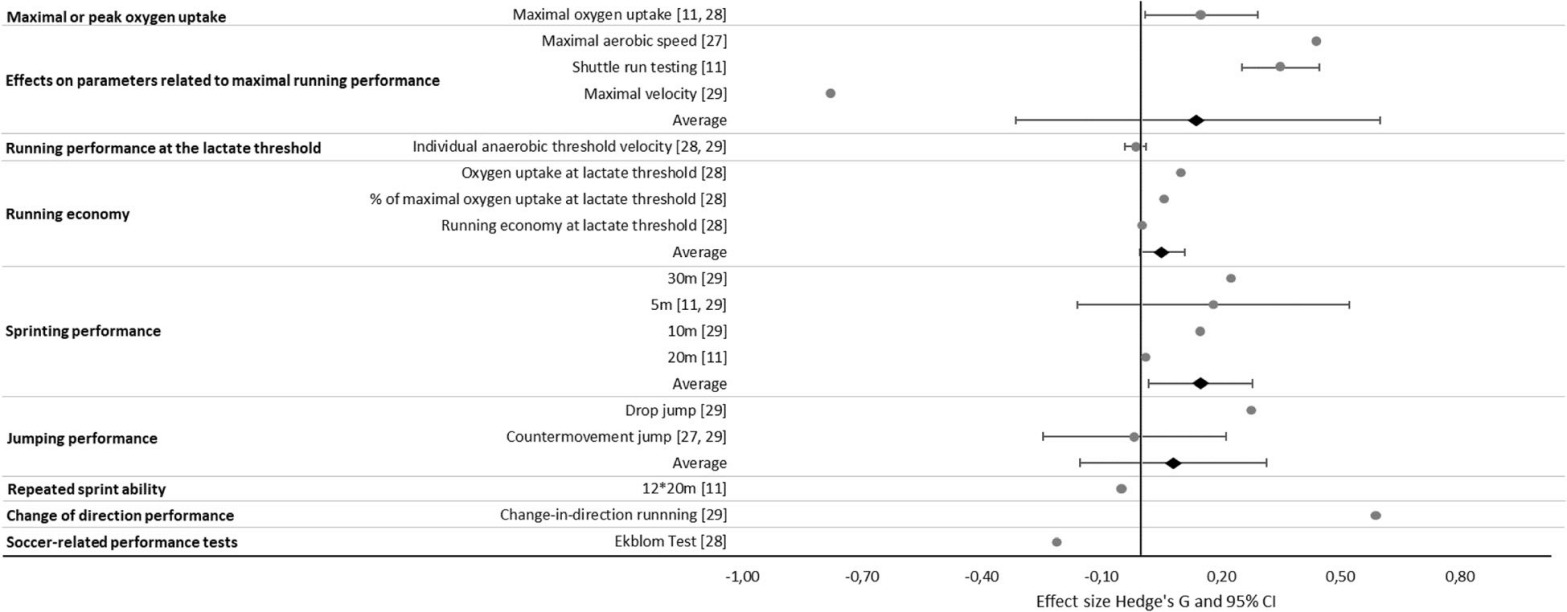
Comparison of the overall and individual effect sizes (*d*_ppc2_, dots) and corresponding 95% confidence intervals (lines), for parameters related to the performance of youth soccer players with HIIT to those of SSG

In comparison to SSG, HIIT exerted a small positive effect on maximal or peak oxygen uptake (mean *d*_ppc2_ 0.15 ± 0.10; range 0.08–0.22), as shown by two studies, one demonstrating a trivial positive effect [28] and the other a small positive effect [11] (Fig. 4).

### Effects on Parameters Related to Maximal Running Performance

#### HIIT Versus All Other Interventions

As assessed in six studies, HIIT induced a moderate mean positive effect on parameters related to maximal running performance (mean *d*_ppc2_ 0.30 ± 0.58; range − 0.78–1.34) [4, 10, 11, 27, 29, 30], in association with either incremental running tests (expressed as maximal aerobic speed (MAS) or *V*_max_) [4, 27, 29], different shuttle run tests [11, 30], or a 1000-m time-trial run [10] (Fig. 2). The *I*^2^ value of 14% indicated low heterogeneity.

#### HIIT Versus All Other Interventions Except SSG

As examined in three studies [4, 10, 30], in comparison to all other interventions except SSG, HIIT induced a large positive mean effect on maximal running performance (mean *d*_ppc2_ 0.55 ± 0.70; range 0.01–1.34) (Fig. 3).

#### HIIT Versus SSG

Three studies [11, 27, 29] demonstrated small positive effects of HIIT on maximal running performance (mean *d*_ppc2_ 0.14 ± 0.52; range − 0.78–0.45) in comparison to SSG, as determined by incremental running tests [27, 29] and shuttle run testing [11] (Fig. 4).

### Running Performance at the Lactate Threshold

#### HIIT Versus All Other Interventions

With respect to running performance at the lactate threshold, reported in four cases, HIIT exerted a large positive mean effect in comparison to all other interventions (mean *d*_ppc2_ 1.00 ± 2.29; range − 0.40–4.42). One of these four studies indicated a moderate negative [4], one trivial negative [28], one no [29], and one large positive effect [13] (Fig. 2). The lactate threshold was determined either from the lactate curve [4, 29] according to the procedure of Stegmann and colleagues [31] or defined as a blood lactate concentration 1.5 mmol/L higher than baseline [13, 28].

#### HIIT Versus All Other Interventions Except SSG

As shown in two reports, in comparison to all other interventions except SSG, HIIT had a large positive effect on running performance at the lactate threshold (mean *d*_ppc2_ 2.01 ± 3.41; range − 0.40–4.42) [4, 13] (Fig. 3).

#### HIIT Versus SSG

Comparison of HIIT to SSG in two publications [28, 29] demonstrated a trivial negative mean effect of the former on running performance at the lactate threshold (mean *d*_ppc2_ − 0.01 ± 0.02; range − 0.03–0.00) (Fig. 4).

### Running Economy

#### HIIT Versus All Other Interventions

In comparison to all other interventions, HIIT induced a large positive mean effect on running economy (mean *d*_ppc2_ 0.53 ± 0.74; range 0.00–1.64). Two trivial positive [28], three small positive [13, 28], and two large positive effects [13] were obtained in the two investigations on different parameters of relevance (i.e., the respiratory exchange ratio, percentage of peak heart rate, and percentage of peak oxygen uptake at the lactate threshold) (Fig. 2). A moderate degree of heterogeneity was indicated by the *I*^2^ value of 43%.

#### HIIT Versus All Other Interventions Except SSG

One article reported that in comparison to all control interventions except SSG, HIIT produced a large positive mean effect on running economy (mean *d*_ppc2_ 0.88 ± 0.83; range 0.10–1.64) [13] (Fig. 3).

#### HIIT Versus SSG

One comparison of HIIT to SSG revealed a trivial positive mean effect of the former on running economy (mean *d*_ppc2_ 0.05 ± 0.05; range 0.00–0.10) [28] (Fig. 4).

### Sprinting Performance

#### HIIT Versus All Other Interventions

HIIT exerted a small positive effect on linear sprinting performance (mean *d*_ppc2_ 0.10 ± 0.12; range − 0.01–0.35) over the various distances (5, 10, 20, 30, and 40 m) examined in five different studies. Trivial effects were found for 5 m [11], 20 m [10, 11, 30], 30 m [10], and 40 m [10]; small positive effects for 10 m [13, 29], 30 m [29], and 40 m [13]; and one moderate positive effect for 5 m [29] (Fig. 2). The *I*^2^ value of 12% indicated low heterogeneity.

#### HIIT Versus All Other Interventions Except SSG

In comparison to all other interventions except SSG, HIIT exerted a trivial positive effect on sprinting performance (mean *d*_ppc2_ 0.06 ± 0.08; range − 0.01–0.17). One small positive effect was found for 10 m [13], two trivial effects for 20 m [10, 30], and one trivial effect each for 30 m [10] and 40 m [10] (Fig. 3).

#### HIIT Versus SSG

In comparison to SSG, HIIT produced a small positive effect on sprint performance (mean *d*_ppc2_ 0.15 ± 0.15; range 0.01–0.35), with one trivial and one moderate positive effect for 5 m [11, 29], one small positive effect for 10 m and 30 m [29], and one trivial positive effect for 20 m [11, 29] (Fig. 4).

### Jumping Performance

#### HIIT Versus All Control Interventions

In comparison to all other interventions, HIIT had a trivial positive effect on jumping performance (countermovement jump, drop jump, squat jump, and vertical jump) (mean *d*_ppc2_ 0.01 ± 0.15; range − 0.14–0.28). Small negative effects were found in three studies [13, 29, 30], and trivial effects were detected in two cases for the countermovement jump [4, 10] and one case each for the drop jump and squat jump [10]. Small positive effects were calculated for the countermovement jump in one investigation [27] and for the drop jump in two studies [4, 29] (Fig. 2). No heterogeneity was observed (*I*^2^ = 0%).

#### HIIT Versus All Other Interventions Except SSG

In comparison to all other interventions except SSG, HIIT showed a trivial negative effect on jumping performance (mean *d*_ppc2_ − 0.02 ± 0.12; range − 0.14–0.22), based on two small negative effects on the vertical jump [13, 30]; four trivial effects, two for the countermovement jump [4, 10] and one each for the drop jump and squat jump [10]; and one small positive effect for the drop jump [4] (Fig. 3).

#### HIIT Versus SSG

In comparison to SSG, HIIT had a trivial positive effect on jumping performance (mean *d*_ppc2_ 0.08 ± 0.21; range − 0.13–0.28), as a result of two small positive effects, one on the countermovement jump [27] and one on the drop jump [29], as well as one small negative effect on the countermovement jump [29] (Fig. 4).

### Repeated Sprint Ability

#### HIIT Versus SSG

Comparison of the impact of HIIT on repeated-sprint ability to that of SSG in one study involving a 12 × 20-m test [11] resulted in a trivial negative effect (*d*_ppc2_ − 0.05) (Figs. 2 and 4).

### Change of Direction Performance

#### HIIT Versus SSG

One publication showed that in comparison to SSG, HIIT exerted a large positive effect on change-of-direction performance (*d*_ppc2_ 0.59) [29] (Figs. 2 and 4).

### Soccer-Related Performance Tests

#### HIIT Versus All Other Interventions

The effect of HIIT on variables related to soccer performance was moderately positive in comparison to that of all other interventions (mean *d*_ppc2_ 0.42 ± 0.42; range − 0.21–1.15) [12, 13, 28] (Fig. 2). The tests involved in this context included technical tests [12, 13, 28], kicking velocity [13], and data collected during actual soccer matches [13] (distance covered, number of sprints, number of contacts with the ball, number of successful and unsuccessful passes). The calculated *I*^2^ of 8% indicated a low degree of heterogeneity.

#### HIIT Versus All Other Interventions Except SSG

Comparison of HIIT to all other interventions except SSG revealed a moderate positive effect of the former on soccer-related performance, including kicking velocity [13], the Loughborough Soccer Passing Test (LSPT) [12], and data collected during actual soccer matches [13] (mean *d*_ppc2_ 0.47 ± 0.39; range − 0.09–1.15) [12, 13] (Fig. 3).

#### HIIT Versus SSG

In comparison to SSG, HIIT exhibited a small negative effect on soccer-related performance (*d*_ppc2_ − 0.21), as assessed by the Ekblom’s test [28] (Fig. 4).

### Time Efficiency

The average durations of one session of HIIT and of all the other programs were 33 ± 14 min and 41 ± 15 min, respectively—a difference that is noteworthy, even if not statistically significant. Considered separately, in the two studies involving high-volume endurance exercise as the control training [4, 10], the average session lasted significantly longer than in the case of HIIT (52 ± 7 min vs 31 ± 3 min). In four studies, the duration of intervention and control sessions was matched [11–13, 27].

## Discussion

The present meta-analysis compares the effects of HIIT on youth soccer players to those of alternative training regimes, including SSG.

The overall findings were as follows:

(i) In comparison to all other interventions, HIIT induces moderate-to-large positive effects on maximal or peak oxygen uptake, variables related to running performance (i.e., maximal running performance, running performance at the lactate threshold and running economy), change-of-direction performance, and soccer-related performance tests (i.e., technical exercises with the ball and game-specific parameters such as the total distance covered, number of sprints, and number of involvements with the ball)
(ii) In comparison to all other interventions except SSG, HIIT demonstrates moderate-to-large effects on maximal or peak oxygen uptake, variables related to running performance, and, again, soccer-related performance
(iii) In comparison to SSG, HIIT exerts a large effect on change-of-direction ability
(iv) In comparison to all other interventions including or excluding SSG, as well as to SSG alone, HIIT has little or no impact on sprint running performance, jumping performance, or repeated sprint ability

Although endurance performance is unquestionably a key determinant of the success of advanced youth soccer players [13, 32], numerous other variables that require time to develop, such as technical and tactical skills, also play major roles [33, 34]. In this context, time-efficient training in the form of HIIT and SSG, especially in comparison to traditional high-volume training, may offer an excellent approach to improving the endurance performance of these athletes. In this regards, HIIT not only takes less time, but also improves VO_2peak_ to a greater extent than other training strategies [35]. Furthermore, intense regimes of this sort involve physiological loads comparable to those encountered in an actual soccer game, where the heart rate averages 85% of HR_max_ and intensities as high as 90–95% of HR_max_ can be reached [2, 36].

In this regards, HIIT and SSG improve endurance parameters (e.g., maximal oxygen uptake, maximal running performance, running performance at the lactate threshold, and running economy) to a similar extent, with slightly higher values of VO_2peak_ for HIIT, in agreement with previous findings [35]. However, SSG also includes soccer-specific drills with the ball or tactical training [14], improving additional determinants of soccer success [19]. Unfortunately, our analysis does not allow definitive determination of which HIIT protocol (sprint training in intervals lasting 15 s to 4 min) and SSG (variations in duration, pitch size, and number of players) are most beneficial to youth soccer players. It is noteworthy that there has been some concern that HIIT may be unpleasant for young athletes [37], as well as may lead to overtraining [38, 39]. Although none of the studies analyzed here reported any signs of overreaching or overtraining, further investigation of the long-term effects of HIIT and SSG is warranted.

The present analysis indicates that improvements in neuromuscular parameters (i.e., sprinting and jumping) with HIIT and SSG were trivial or, at best, small. In most cases, HIIT involved 4-min intervals at 90–95% of peak heart rate, but not sprinting for only a few seconds at 100% intensity [40], and the same was true for SSG. In this context, the most pronounced positive effects on sprinting and jumping performance were found in two studies using short (15–30 s) intervals of HIIT [4, 29], indicating that for improving sprinting, intervals less than approximately 15 s may be more beneficial. With the constantly increasing demands on the sprinting ability of youth soccer players from under 13 to younger than 18 years of age [41], the development of explosive strength, often decisive for success, appears to be one major aspect for further development. As also indicated by the effects of HIIT and SSG concerning sprinting and jumping described here, additional training of these abilities appears to be beneficial for youth soccer players. Furthermore, when developing neuromuscular abilities, the state of the child’s maturation should be taken into consideration and before puberty, development of strength and speed should be the main target [42]. In adolescents, additional components such as power and hypertrophy should be developed [42].

The single investigation that examined repeated sprints [11] observed a trivial effect of HIIT in comparison to SSG, i.e., SSG and HIIT appear to improve this ability to a similar extent. From a practical point of view, shorter intervals of HIIT or SSG at intensities close to that of repeated sprints may be sufficient to improve such performance. In the one investigation on change-of-direction performance [29], HIIT had a large positive effect in comparison to SSG, in contrast to other findings of greater improvement of this sort with SSG [43], a discrepancy that cannot be explained at present. The effects on repeated sprint ability and change of direction performance both require further scientific evaluation.

In practice, coaches may focus on the effects of various training regimes on performance in connection with soccer-related tests. The medium-positive effect of HIIT on such performance compared to all other interventions can be attributed to the large positive effects on the number of sprints, total distance covered, and number of involvements with the ball, all of which require the endurance that can be achieved with HIIT. The negative effect of HIIT in the Ekblom’s test in comparison to SSG (Fig. 4) may be explained by the various soccer-specific movements involved in this test (i.e., jumping, slalom running, running backward, changes in direction, running sideways), which are most closely mimicked in SSG.

HIIT and SSG provide similar benefits with respect to most of the parameters analyzed here (Fig. 4). Moreover, SSG may offer additional advantages, improving essential neuro-muscular and cognitive skills such as reaction time, decision-making, and change-of-direction speed [44]. Participants may also experience greater motivation [45] and enjoyment [27] when performing SSG than HIIT protocols that are less sport-specific. At the same time, the intensity of SSG can be varied greatly, being higher on larger pitches and with a smaller number of players [16], and the optimal design remains to be determined.

From a practical point of view, the choice of a HIIT or SSG protocol for training youth soccer players depends to a large extent on the time-point during the season and the coach’s overall strategy. HIIT induces somewhat more pronounced increases in VO_2peak_ than SSG, thereby developing adequate aerobic endurance. During a season, running-based protocols that do not involve ball drills often appear impractical. From this perspective, SSG may provide a viable alternative for maintaining endurance and skills such as ball handling and tactical thinking.

## Limitations

The following limitations of the present analysis need to be considered: (1) Only nine studies met our criteria for inclusion, and in some cases, no more than one ES could be calculated for each parameter; (2) for some parameters, there was considerable heterogeneity (as indicated by the 95% CI), especially for running performance at the lactate threshold; (3) we categorized each report on the basis of what the authors stated. For instance, in one of the studies [11] involving repeated sprints as the intervention, the authors stated explicitly that these were performed as a form of HIIT; (4) we cannot assess the benefits of HIIT and SSG to youth soccer players in individual competitive situations, since a standardized soccer match (or simulation thereof) does not yet exist; (5) there may be major age-dependent differences in the development of the parameters investigated here, and there is no clear-cut division between youth and adult soccer players; (6) although all of the HIIT and SSG protocols analyzed met our criteria, these protocols were somewhat heterogeneous, especially with respect to duration, intensity, and ratio of load to rest (Table 2).

## Conclusions

On the basis of the present analysis, we conclude that HIIT in general and the more sport-specific SSG both result in similar improvements in maximal or peak oxygen uptake, variables related to running performance (i.e., maximal running performance, running performance at the lactate threshold, and running economy), and soccer-related performance tests in youth players. In contrast, repeated sprint ability and sprinting and jumping performance are virtually unaffected by either HIIT or SSG. Change of direction performance was improved to a greater extent by HIIT than SSG, but this difference requires further investigation. Thus, HIIT and SSG both offer an effective approach to simultaneously improve certain physiological characteristics and the sport-specific skills of youth soccer players.

## Abbreviations

AR: Active rest
CI: Confidence interval
CinD: Change-in-direction run
CMJ: Countermovement jump
DJ: Drop jump
ES: Effect size
HIIT: High-intensity interval training
HR_max_: Maximal heart rate
HR_peak_: Peak heart rate
HVT: High-volume training
IAT: Individual anaerobic threshold
Lac_max_: Maximal blood lactate concentration
LIST: Loughborough Intermittent Shuttle Test
LSPT: Loughborough Soccer Passing Test
LT: Lactate threshold
MAS: Maximal aerobic speed
MSFT: Multistage fitness test
n.i.: Not indicated
PEDro: Physiotherapy Evidence Database
PR: Passive rest
RE: Running economy
Reps: Repetitions
RSA: Repeated sprint ability
SJ: Squat jump
SSG: Small-sided game
TT: Technical training
TTE: Time to exhaustion
VJ: Vertical jump
*V*_max/peak_: Maximal velocity during incremental step test
VO_2max_: Maximal oxygen uptake
VO_2peak_: Peak oxygen uptake
VOT: Volume-oriented training at low intensities
VT: Ventilatory threshold
YYIETL1: Yo-Yo intermittent endurance test level 1
YYIRTL1: Yo-Yo intermittent recovery test level 1
